# Reduced isolation-induced pup ultrasonic communication in mouse pups lacking brain serotonin

**DOI:** 10.1186/s13229-015-0003-6

**Published:** 2015-03-08

**Authors:** Valentina Mosienko, Daniel Beis, Natalia Alenina, Markus Wöhr

**Affiliations:** Molecular Biology of Peptide Hormones, Max-Delbrück-Center for Molecular Medicine, Robert-Rössle-Str. 10, D-13125 Berlin, Germany; School of Physiology and Pharmacology, University of Bristol, University Walk, BS8 1TD Bristol, UK; St. Petersburg State University, 7-9, Universitetskaya nab, St. Petersburg, 199034 Russia; Behavioral Neuroscience, Experimental and Biological Psychology, Philipps-University of Marburg, Gutenbergstr. 18, D-35032 Marburg, Germany

**Keywords:** Animal models, Serotonin, Neurodevelopmental disorders, Autism, Communication, Ultrasonic vocalizations

## Abstract

**Background:**

Serotonin (5-hydroxytryptamine, 5-HT) is a key modulatory neurotransmitter in the mammalian central nervous system (CNS) that plays an important role as a developmental signal. Several lines of evidence associate altered 5-HT signaling with psychopathology in humans, particularly neurodevelopmental disorders such as autism spectrum disorders (ASD). ASD are characterized by persistent social and communication deficits along with stereotyped and repetitive patterns of behavior, with all symptoms emerging early during development.

**Methods:**

Here, we employed a mouse model devoid of brain 5-HT due to the lack of the gene encoding tryptophan hydroxylase 2 (*Tph2*), the initial and rate-limiting enzyme of 5-HT synthesis in the CNS. *Tph2* null mutant (*Tph2*^*-/-*^) mice show normal prenatal development; however, they display for yet unknown reasons severe growth retardation during the first postnatal weeks. We investigated, therefore, whether *Tph2*^*-/-*^ mice display deficits in isolation-induced ultrasonic vocalizations (USV) as pups during early life. Isolation-induced USV are the most commonly studied behavioral measure to assess developmental delays and communication deficits in rodent models for ASD, particularly as they serve an important communicative function in coordinating mother-pup interactions.

**Results:**

*Tph2*^*-/-*^ mouse pups displayed a clear deficit in the emission of isolation-induced USV, as compared to heterozygous and wildtype littermates, exactly during growth retardation onset, including reduced call numbers and deficits in call clustering and temporal organization.

**Conclusions:**

The ultrasonic communication impairment displayed by *Tph2*^*-/-*^ mouse pups is likely to result in a deficient mother-infant interaction, presumably contributing to their growth retardation phenotype, and represents a prominent feature relevant to ASD.

## Background

Serotonin (5-hydroxytryptamine, 5-HT) is a key modulatory neurotransmitter in the mammalian central nervous system (CNS; [1]). The 5-HT system is one of the earliest developing neurotransmitter systems, with 5-HT itself and expression of associated enzymes and receptors all reaching peak levels during early brain development before declining to adult levels, reflecting its important role as a developmental signal [2,3]. It is, therefore, not surprising that several lines of evidence associate altered 5-HT signaling with psychopathology in humans, particularly neurodevelopmental disorders such as autism spectrum disorders (ASD; [4]).

ASD are characterized by persistent social and communication deficits along with stereotyped and repetitive patterns of behavior, with all symptoms emerging early during development [5]. In rodent models for ASD, however, behavioral phenotypes with relevance to the diagnostic criteria are typically assessed in adulthood, since few behavioral test paradigms are available that allow the reliable assessment of such complex behavioral alterations in infancy [6,7]. Yet, a remarkable exception appears to be the experimentally controlled induction of ultrasonic vocalizations (USV) in infant rodents during isolation from mother and littermates for a short period of time. Such isolation-induced USV are commonly studied to assess developmental delays and communication deficits in rodent models for ASD, with model organisms typically displaying overall reduced USV levels or an ontogenetic shift in the inverted U-shaped developmental call emission pattern [8-10].

The involvement of the 5-HT system in isolation-induced pup USV has been extensively investigated in pharmacological studies. Diminishing central 5-HT levels by means of the neurotoxin 5,7 dihydroxytryptamine (5,7-DHT) or the inhibitor of the 5-HT synthesizing enzyme tryptophan hydroxylase (TPH), para-chlorophenylalanine (PCPA), led to a strong reduction in USV emission, with PCPA effects being dose-dependently antagonized by the 5-HT precursor 5-hydroxytryptophan (5-HTP; [11]). Consistently, the long-term reduction in 5-HT content following repeated MDMA treatment was also found to reduce USV emission rates [12]. Furthermore, inhibiting 5-HT function by the application of 8-OH-DPAT, which works mostly through activation of the 5-HT1A autoreceptor [13], led to an inconsistent result pattern in mouse pups [14,15], but dose-dependently decreased isolation-induced USV in rat pups in a comparatively consistent manner [12,16-23]. However, not only decreasing 5-HT function, but also acutely increasing extracellular 5-HT levels by selective serotonin reuptake inhibitors (SSRI) reduces isolation-induced USV in mice [24] and rats [20,21,25-29]. Likewise, 5-HT release induced by acute MDMA treatment also results in the inhibition of isolation-induced USV [12].

While the pharmacological approach strongly suggests the involvement of 5-HT in isolation-induced USV, this approach has a number of limitations, possibly accounting for at least some of the obtained inconsistencies: (1) 5,7-DHT treatment leads to the lesion of 5-HTergic neurons, also affecting 5-HT cotransmitter levels; thus, observed effects might not exclusively be due to the lack of 5-HT *per se*; (2) PCPA does not only inhibit the synthesis of central 5-HT, but also alters its synthesis in the periphery; (3) both 5,7-DHT and PCPA cause only a partial decrease in 5-HT content; (4) 5-HT receptor agonists/antagonists are typically not selective for a specific receptor subtype and might also activate/inhibit non-5-HT receptors; finally, (5) at least some of the observed effects on isolation-induced USV following the administration of 5-HT receptor ligands might be unspecific and actually be caused by their sedative or thermoregulatory actions [9].

In the present study, we aimed to overcome the limitations of the pharmacological approach in studying the impact of brain 5-HT on isolation-induced USV by means of a genetic mouse model lacking the central form of TPH, TPH2 [30], and, thus, genetically depleted of central 5-HT [31]. Previous studies showed that *Tph2* null mutant mice not only exhibit severe growth retardation during early development [31-35], but also a number of behavioral alterations in adulthood, most notably in the emotional domain, being characterized by decreased anxiety-related behavior, but enhanced impulsivity [36,37]. In addition, very prominent deficits were seen in the social domain, with a lack of maternal care [31,38], strongly increased aggression [31,34,36,37,39], and deficits in sexual behavior [40,41]. In fact, a recent study suggested that *Tph2* null mutant mice display deficits with relevance to all ASD core symptoms, including sociability, scent marking, and repetitive behavior, while acoustic communication was not assessed [33]. Here, we investigated, therefore, whether *Tph2* null mutant mouse pups display deficits in isolation-induced USV, the most commonly studied behavioral measure to assess developmental delays and communication deficits in rodent models for ASD.

## Methods

### Ethics approval

All procedures were conducted in strict compliance with the National Institutes of Health Guidelines for the Care and Use of Laboratory Animals and the legal requirements of Germany. Procedures were approved by the ethical committee of the local government (Regierungspräsidium, Gießen, Germany).

### Animals and housing

Isolation-induced pup USV were assessed in *Tph2* null mutant (*Tph2*^*-/-*^) mice with a targeted deletion of exon 1 and 2 in the *Tph2* gene and compared to *Tph2* heterozygous (*Tph2*^*+/-*^) and *Tph2* wildtype (*Tph2*^*+/+*^) mice. Mice were obtained from mutant lines originally generated by Alenina et al. [31] and backcrossed to C57BL/6N (Charles River, Sulzfeld, Germany; F10 generation; [37]). Using a heterozygous breeding protocol, *Tph2*^*+/-*^ males and females were paired for breeding in a conventional vivarium at the Biomedical Research Center of the Philipps-University of Marburg, Germany. Approximately 2 weeks after pairing for breeding, females were individually housed and inspected daily for pregnancy and delivery. The day of birth was considered as postnatal day (PND) 0. In total, pups from seven litters were included in the experiment. In all litters included, all three genotypes were present, namely *Tph2*^*-/-*^, *Tph2*^*+/-*^, and *Tph2*^*+/+*^ littermates. Bedding and a wooden board were provided in each cage. Standard rodent chow and water were available *ad libitum*. The colony room was maintained on a 12:12 light/dark cycle with lights on at 06:00 h, at approximately 22°C and 40% to 50% humidity. Pups were identified by paw tattoo, using non-toxic animal tattoo ink (Ketchum permanent Tattoo Inks green paste, Ketchum Manufacturing Inc., Brockville, Canada). The ink was inserted subcutaneously through a 30-gauge hypodermic needle tip into the center of the paw, as in previous studies [42,43].

### Genotyping

Mouse tail snips were collected by dissecting approximately 0.3 cm of tail. Tails were digested in a buffer containing 0.2 SDS and 1 mg/ml proteinase K, diluted in TE buffer containing 20 μg/ml RNase A and subsequently used as a DNA template for PCR reaction with the following primers: TPH34 (5′-AGC TGA GGC AGA CAG AAA GG-3′), TPH54 (5′-CCA AAG AGC TAC TCG ACC TAC G-3′), and Neo3 (5′-CTG CGC TGA CAG CCG GAA CAC-3′).

### Behavioral testing

To study developmental aspects in isolation-induced pup USV in a genotype- and sex-dependent manner, an experimental design with three independent variables was used, namely genotype, sex, and development. To this aim, male and female *Tph2*^*-/-*^, *Tph2*^*+/-*^, and *Tph2*^*+/+*^ littermates were repeatedly tested on PND3, PND6, and PND9. Recordings of isolation-induced USV were conducted during the light phase of the 12:12 h light/dark cycle. After completion of behavioral experiments on PND3, pups were tattooed for their identification and tail samples were taken for genotyping. Experimenters were blind to genotypes during data acquisition and analysis.

#### Isolation-induced pup ultrasonic vocalizations - recording

To induce isolation-induced USV, pups were isolated from mother and littermates for 10 min under room temperature (20°C to 23°C). Pups were individually removed from the nest at random and gently placed into an isolation container (10 × 8 × 6 cm; open surface) made of glass, containing fresh bedding material. The isolation container was surrounded by a sound attenuating box (21 × 21 × 21 cm) made of Styrofoam (thickness of walls: 6 cm). USV emission was monitored by an UltraSoundGate Condenser Microphone CM 16 (Avisoft Bioacoustics, Berlin, Germany) placed in the roof of the sound attenuating box, 22 cm above the floor. The microphone was connected via an UltraSoundGate 416 USGH audio device (Avisoft Bioacoustics) to a personal computer, where acoustic data were recorded with a sampling rate of 250,000 Hz in 16-bit format by Avisoft RECORDER (version 2.97; Avisoft Bioacoustics). The microphone that was used for recording was sensitive to frequencies of 15 to 180 kHz with a flat frequency response (±6 dB) between 25 and 140 kHz. Prior to each test, the behavioral equipment was cleaned using a 0.1 % acetic acid solution and dried with paper towels.

#### Isolation-induced pup ultrasonic vocalizations - analysis

For acoustical analysis, recordings were transferred to Avisoft SASLab Pro (version 5.20; Avisoft Bioacoustics), and a fast Fourier transform was conducted (512 FFT length, 100% frame, Hamming window, and 75% time window overlap). Correspondingly, the spectrograms were produced at 488 Hz of frequency resolution and 0.512 ms of time resolution. Call detection was provided by an automatic threshold-based algorithm (amplitude threshold: −40 dB) and a hold-time mechanism (hold time: 10 ms). Since no USV were detected below 30 kHz, a high-pass filter of 30 kHz was used to reduce background noise outside the relevant frequency band to 0 dB. The accuracy of call detection by the software was verified manually by an experienced user. When necessary, missed calls were marked by hand to be included in the automatic parameter analysis by trained experimenters. The total number of USV was calculated for the entire 10-min test session. Based on previous studies of isolation-induced pup USV [42-44], latency to start calling, call duration, peak frequency, peak amplitude, and frequency modulation were also included (for details, see [42]). In addition, call subtypes were determined by means of density blots depicting peak frequency versus peak amplitude, as described before [10]. Finally, to assess the temporal organization of isolation-induced USV emission, sequential analyses were performed by correlating the durations of given isolation-induced USV with the durations of the previous ones (*N* − 1), the ones two before (*N* − 2), and the ones three before (*N* − 3), as described before [10].

#### Developmental milestones and somatosensory reflexes

In addition to isolation-induced USV, developmental milestones and somatosensory reflexes were assessed by trained experimenters on PND3, PND6, and PND9. After the 10-min isolation period, body weight, body temperature, surface righting, and vertical screen holding were determined as described in Wöhr et al. [42], with a maximum latency of 30 s for surface righting and vertical screen holding. Body weight was measured using a palmscale (PS6-250; My Weigh Europe, Hückelhoven, Germany). For body temperature determination, a Testo 110 thermometer with surface sensor (Testo AG, Lenzkirch, Germany) was used. Body temperature was measured by gentle application of the thermal probe onto the stomach of the mouse pup for 20 s.

### Statistical analysis

For analysis of isolation-induced USV, developmental milestones, and somatosensory reflexes, ANOVAs for repeated measurements with the between-subjects factors genotype and sex, and the within-subject factor development were calculated. Litter was used as covariate. Approximately half of the *Tph2*^*+/-*^ mice were randomly excluded from the experiment to obtain similar numbers of mice per genotype in order to improve the quality of statistical comparisons, resulting in the following group sizes: 10 *Tph2*^*-/-*^, 11 *Tph2*^*+/-*^, and 12 *Tph2*^*+/+*^ littermates. ANOVAs were followed by LSD *post hoc* analysis when appropriate. A *P* value of < .050 was considered statistically significant. The covariate litter never reached statistical significance (all *P* values > .050).

## Results

To evaluate whether isolation-induced pup USV are altered in the absence of brain 5-HT in mice, we evaluated USV at three developmental stages, namely PND3, PND6, and PND9, and analyzed developmental, genotype, and sex effects in the USV emission pattern of *Tph2*^*-/-*^*, Tph2*^*+/-*^, and *Tph2*^*+/+*^ littermates.

### Isolation-induced pup ultrasonic vocalizations - development

**Figure 1:**
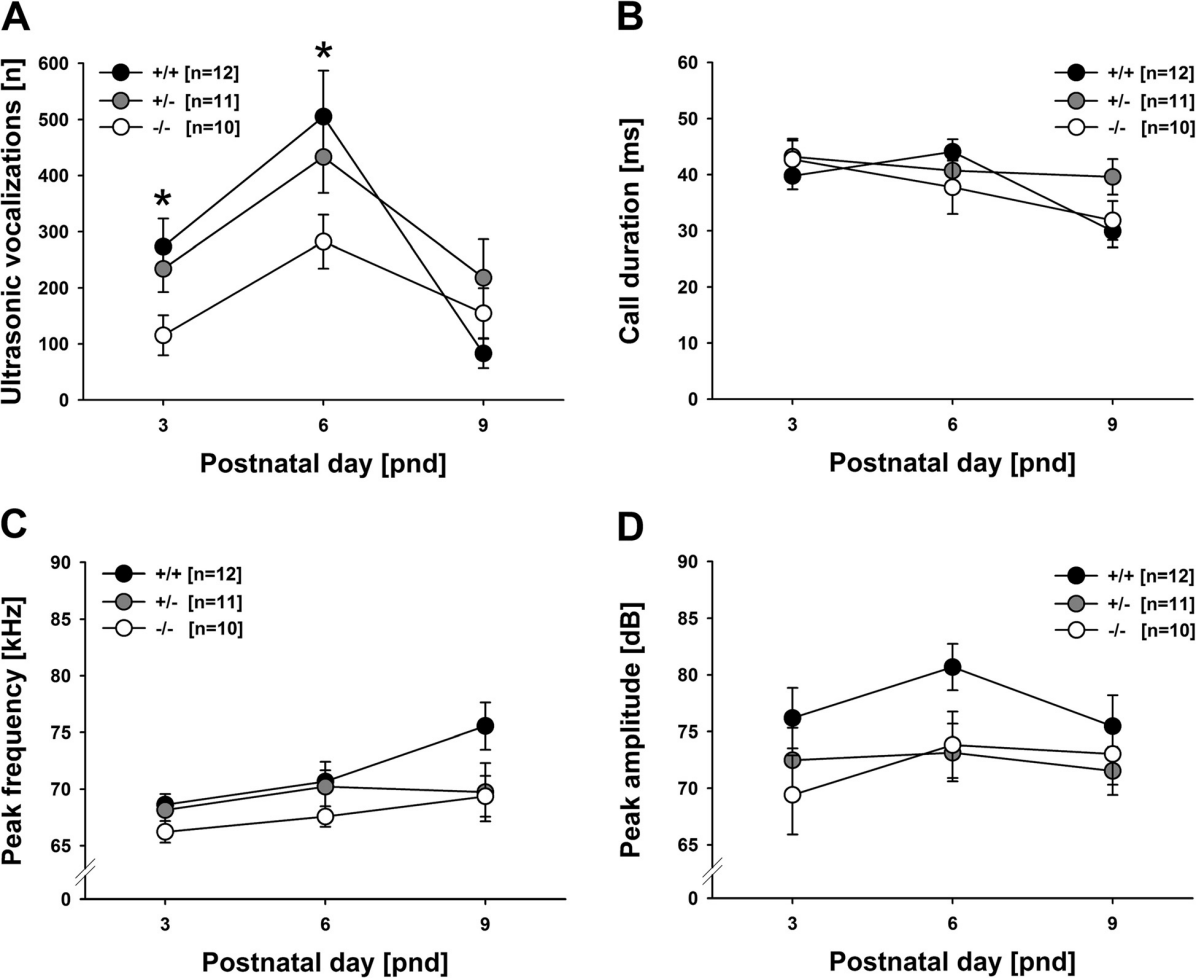
**Isolation-induced ultrasonic vocalizations (USV) in** ***Tph2*** ^***-/-***^ **mouse pups emitted at postnatal days (PND) 3, 6, and 9. (A)** Total number (*n*), **(B)** average call duration in milliseconds (ms), **(C)** average call peak frequency in kilohertz (kHz), and **(D)** average call peak amplitude in decibel (dB) of isolation-induced USV emitted during the 10-min isolation from mother and littermates. Black circles: *Tph2* wildtype (*Tph2* ^*+/+*^) control mice; grey circles: *Tph2* heterozygous (*Tph2* ^*+/-*^) mice; white circles: *Tph2* null mutant (*Tph2* ^*-/-*^) mice. Data are presented as means ± standard errors of the mean. *P < .050 *Tph2* ^*+/+*^ vs. *Tph2* ^*-/-*^.

Emission rates of isolation-induced USV changed with development (main effect development: *F*_2,52_ = 13.916, *P* < .001; Figure 1A), when including all genotypes in the analysis. Specifically, an inverted U-shaped developmental call emission pattern was detected, with call emission rates peaking on PND6 but comparatively low levels of isolation-induced USV on PND3 and PND9. Call duration also changed with development (main effect development: *F*_2,42_ = 3.428, *P* = .042; Figure 1B). Yet, its developmental profile markedly differed from call number as it was characterized by a gradual decrease, with particularly short isolation-induced USV on PND9. Latency to start calling, peak frequency, peak amplitude, and frequency modulation did not change with development (all *P* values > .050; Figure 1C,D).

### Isolation-induced pup ultrasonic vocalizations - genotype

The developmental emission pattern of isolation-induced USV differed between genotypes (main effect genotype: *F*_2,26_ = 2.700, *P* = .086; interaction genotype × development: *F*_4,52_ = 3.526, *P* = .013; Figure 1A). On PND3, *Tph2*^*-/-*^ mouse pups emitted fewer isolation-induced USV than *Tph2*^*+/+*^ controls (*P* = .016) and tended to emit fewer than *Tph2*^*+/-*^ mouse pups (*P* = .069), with the latter not differing from each other (*P* = .517). Similarly, on PND6, again *Tph2*^*-/-*^ mouse pups emitted fewer isolation-induced USV than *Tph2*^*+/+*^ controls (*P* = .024), but did not differ from *Tph2*^*+/-*^ mouse pups (*P* = .125), with the latter also not differing from each other (*P* = .434). On PND9, genotypes did not differ from each other (all *P* values > .050). Importantly, when including maximum one pup per genotype and sex per litter by randomly excluding the additional ones, a very similar result pattern was obtained. Again, the developmental emission pattern of isolation-induced USV differed between genotypes (main effect genotype: *F*_2,19_ = 1.986, *P* = .165; interaction genotype × development: *F*_4,38_ = 3.340, *P* = .021). On PND3 and PND6, *Tph2*^*-/-*^ mouse pups emitted fewer isolation-induced USV than *Tph2*^*+/+*^ controls (*P* = .034 and *P* = .031; all other *P* values > .050), consistent with the statistical analysis including additional littermates. Also in line with it, on PND9, genotypes did not differ from each other (all *P* values > .050). As no genotype effects on the latency to start calling (main effect genotype: *F*_2,26_ = .379, *P* = .689; interaction genotype × development: *F*_4,52_ = .691, *P* = .601) and call duration (main effect genotype: *F*_2,21_ = .339, *P* = .717; interaction genotype × development: *F*_4,42_ = 2.204, *P* = .085; Figure 1B) were detected, this indicates that genotype primarily affected call repetition rate, i.e., numbers of isolation-induced USV per time interval. Peak frequency, peak amplitude, and frequency modulation were not affected by genotype (all *P* values > .050; Figure 1C,D).

### Isolation-induced pup ultrasonic vocalizations - sex

Sex had only a minor effect on the emission of isolation-induced USV. Specifically, a sex difference was obtained for call duration, with females emitting calls with longer durations than males, irrespective of genotype (main effect sex: *F*_1,21_ = 5.773, *P* = .026; interaction sex × development: *F*_2,42_ = 3.058, *P* = .058; interaction genotype × sex: *F*_2,21_ = 2.701, *P* = .090; interaction genotype × sex × development: *F*_4,42_ = 2.221, *P* = .083). Importantly, while in males a gradual decrease in call duration with age was observed, call duration was highest on PND6 in females, reflecting an inverted U-shaped developmental pattern. No evidence for sex differences was obtained for the latency to start calling, call number, peak frequency, peak amplitude, and frequency modulation (all *P* values > .050).

### Isolation-induced pup ultrasonic vocalizations - detailed analyses

**Figure 2:**
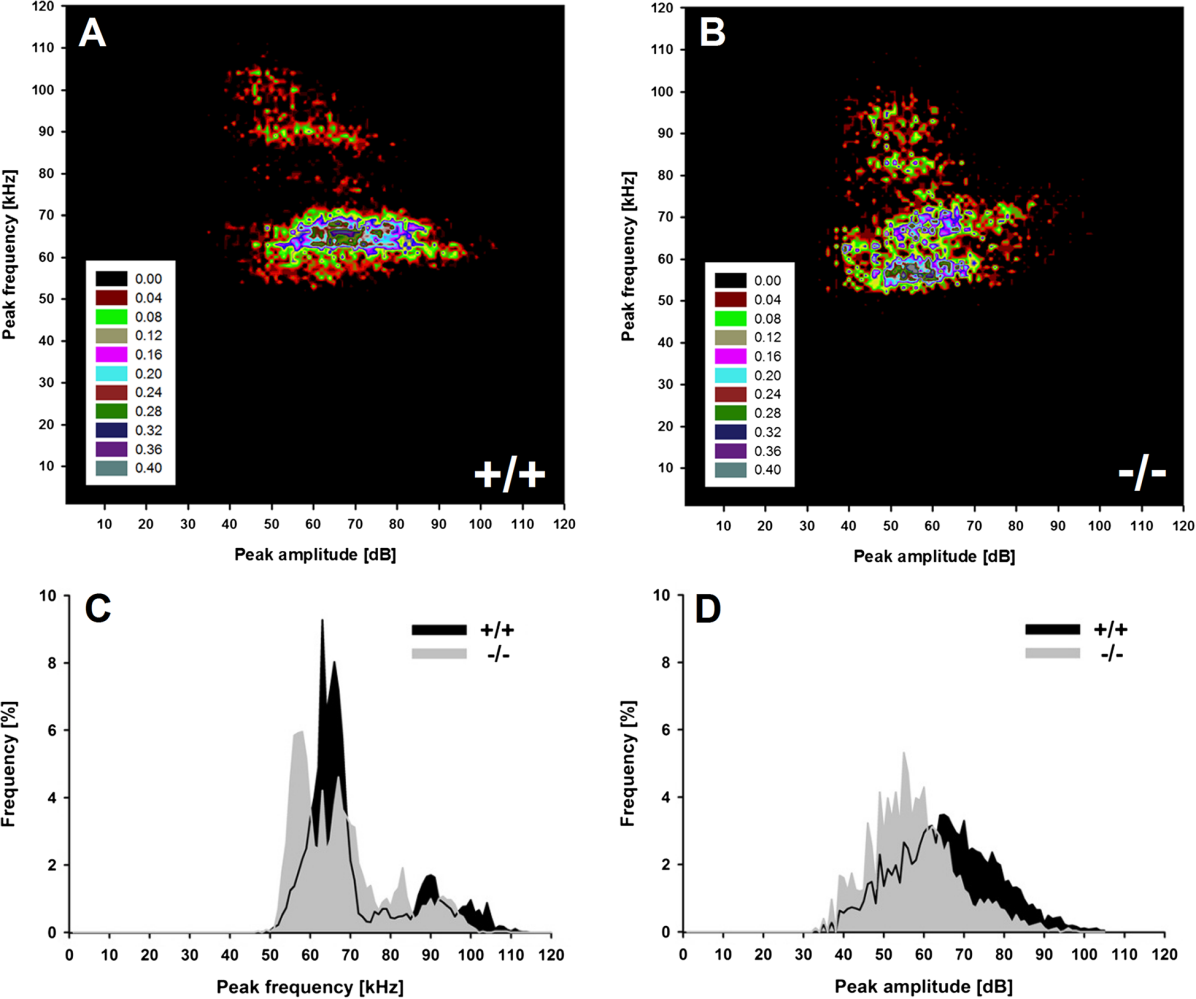
**Distribution of individual isolation-induced ultrasonic vocalizations (USV) in** ***Tph2*** ^***-/-***^ **mouse pups emitted at postnatal day (PND) 6.** Density plots depicting the distribution of individual isolation-induced USV depending on call peak frequency in kilohertz (kHz) and call peak amplitude in decibel (dB) in *Tph2* wildtype (*Tph2* ^*+/+*^) control mice **(A)** and *Tph2* null mutant (*Tph2* ^*-/-*^) mice **(B)**, with color coding reflecting frequencies as percentages. Frequency histograms depicting the distribution of individual USV depending on call peak frequency in kilohertz (kHz) **(C)** and call peak amplitude in decibel (dB) **(D)** in percentages, with isolation-induced USV emitted by *Tph2* wildtype (*Tph2* ^*+/+*^) control mice (black area) and *Tph2* null mutant (*Tph2* ^*-/-*^) mice (grey area).

**Figure 3:**
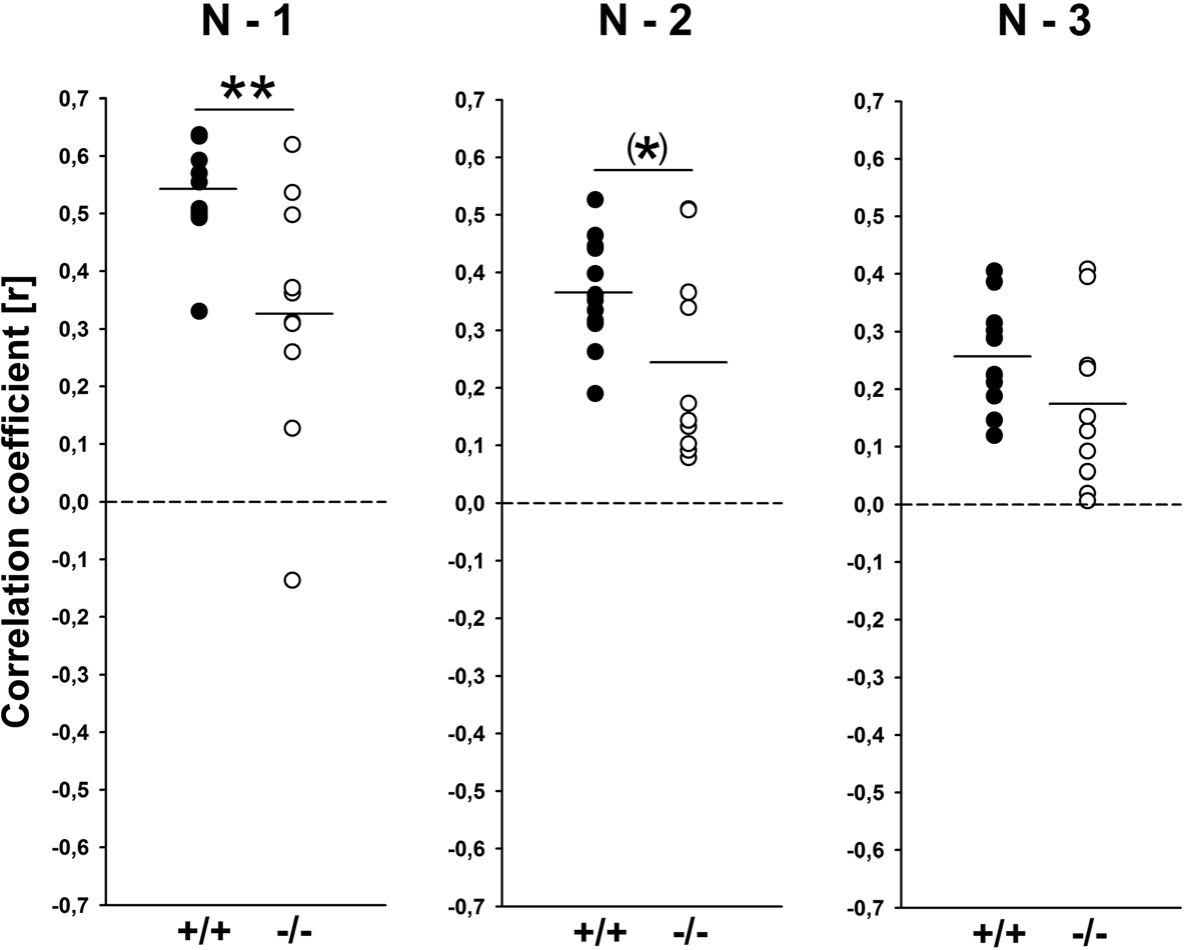
**Sequential analysis of the durations of subsequent isolation-induced ultrasonic vocalizations (USV) indicating a non-random call emission pattern in** ***Tph2*** ^***-/-***^ **mouse pups at postnatal day (PND) 6.** Correlations between the call durations of given isolation-induced USV and the call durations of the previous ones (*N* − 1), the call durations of the ones two before (*N* − 2), or the call durations of the ones three before (*N* − 3) for *Tph2* wildtype (*Tph2* ^*+/+*^) control mice (black circles) and *Tph2* null mutant (*Tph2* ^*-/-*^) mice (white circles). *P < .100 *Tph2* ^*+/+*^ vs. *Tph2* ^*-/-*^; **P < .005 *Tph2* ^*+/+*^ vs. *Tph2* ^*-/-*^.

A more detailed analysis was performed to identify clusters of isolation-induced USV emitted by *Tph2*^*-/-*^ mouse pups and *Tph2*^*+/+*^ controls by means of density plots (Figure 2A,B). For this subsequent analysis, isolation-induced USV emitted on PND6 were used, as an inverted U-shaped developmental call emission pattern was detected in the first step, with call emission rates peaking on PND6, but comparatively low levels of isolation-induced USV on PND3 and PND9. In *Tph2*^*+/+*^ controls, two clusters were identified, with most isolation-induced USV either being characterized by peak frequencies between 60 to 70 and 80 to 110 kHz. Two very similar clusters were obtained in *Tph2*^*-/-*^ mouse pups. However, the two clusters were not as clearly segregated as in *Tph2*^*+/+*^ controls, and the distribution of isolation-induced USV was affected by genotype. Furthermore, in *Tph2*^*-/-*^ mouse pups, the most common peak frequency was below 60 kHz and therefore clearly lower than the most common peak frequency in *Tph2*^*+/+*^ controls, as also evident from peak frequency histograms (Figure 2C). In fact, when comparing peak frequencies between *Tph2*^*-/-*^ mouse pups and *Tph2*^*+/+*^ controls on PND6 only, genotypes differed from each other (*t*_20_ = 2.275, *P* = .034). Also, a substantial number of isolation-induced USV emitted by *Tph2*^*-/-*^ mouse pups were characterized by comparatively low peak amplitudes (Figure 2D), resulting in a trend for louder isolation-induced USV in *Tph2*^*+/+*^ controls, as compared to *Tph2*^*-/-*^ mouse pups on PND6 (*t*_20_ = 1.968, *P* = .063). An additional sequential analysis of the durations of subsequent isolation-induced USV finally indicated that the call emission pattern is not random in *Tph2*^*+/+*^ controls, since the durations of given isolation-induced USV could be predicted by the durations of the previous ones (*N* − 1) and by the ones two before by trend (*N* − 2), but not by the ones three before (*N* − 3). Evidence for such a non-random call emission pattern was also obtained in *Tph2*^*-/-*^ mouse pups but in fewer cases. While substantial correlations of *r* > 0.200 between given isolation-induced USV and the durations of the previous ones (*N* − 1) were found in 100% of the *Tph2*^*+/+*^ controls, such correlations were found in only 80% of the *Tph2*^*-/-*^ mouse pups. Furthermore, correlation coefficients were found to be lower in *Tph2*^*-/-*^ mouse pups, as compared to *Tph2*^*+/+*^ controls (*N* − 1: *t*_20_ = 3.146, *P* = .005; *N* − 2: *t*_20_ = 2.025, *P* = .063; *N* − 3: *t*_20_ = 1.698, *P* = .105; Figure 3).

### Developmental milestones and somatosensory reflexes - development

**Figure 4:**
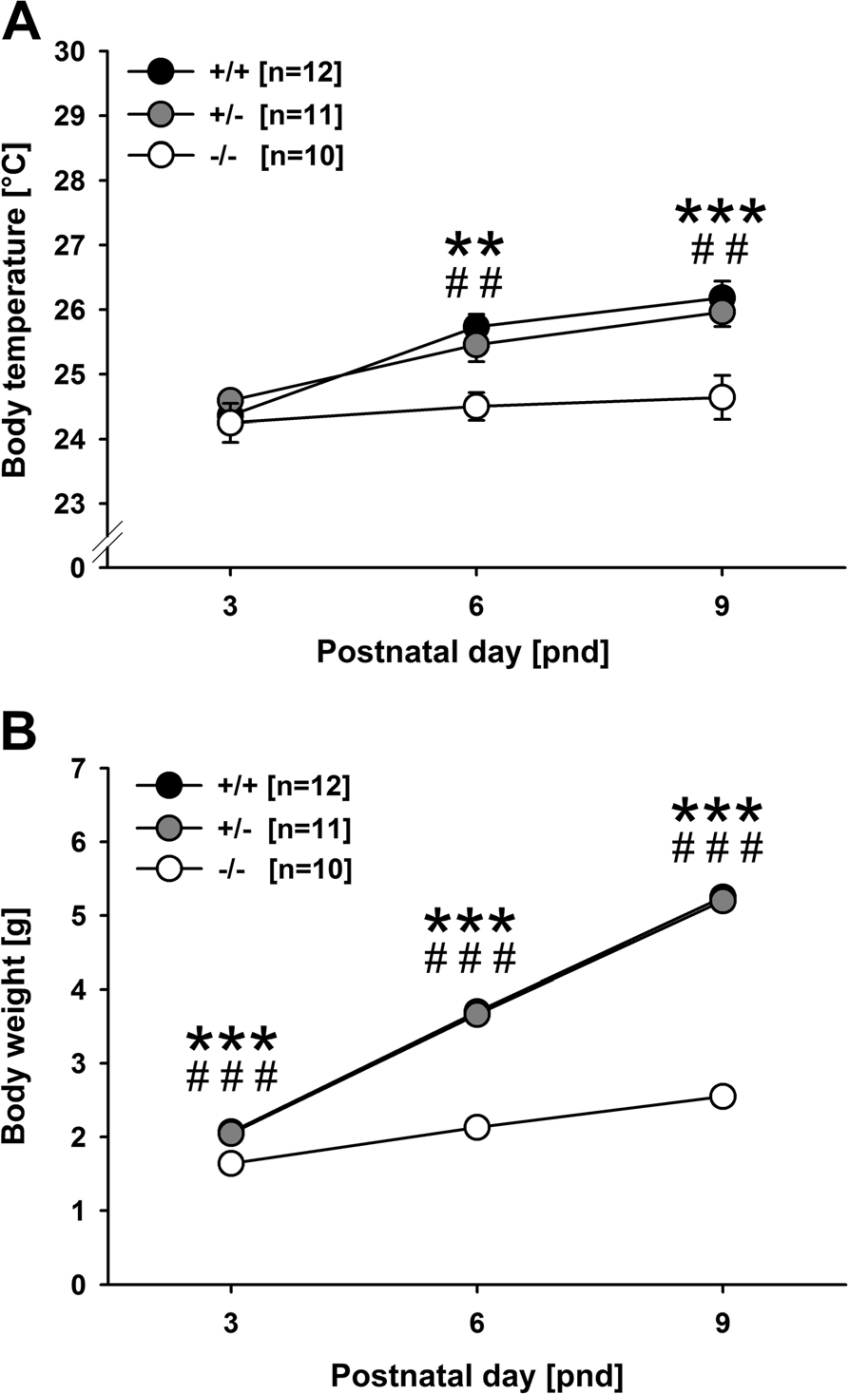
**Developmental profile of** ***Tph2*** ^***-/-***^ **mouse pups at postnatal days (PND) 3, 6, and 9. (A)** Body temperature in degrees Celsius (°C) and **(B)** body weight in grams (g) in pups tested for isolation-induced ultrasonic vocalizations. Black circles: *Tph2* wildtype (*Tph2* ^*+/+*^) control mice; grey circles: *Tph2* heterozygous (*Tph2* ^*+/-*^) mice; white circles: *Tph2* null mutant (*Tph2* ^*-/-*^) mice. Data are presented as means ± standard errors of the mean. **P < .005 *Tph2* ^*+/+*^ vs. *Tph2* ^*-/-*^; ***P < .001 *Tph2* ^*+/+*^ vs. *Tph2* ^*-/-*^; ^##^ *P* < .005 *Tph2* ^*+/-*^ vs. *Tph2* ^*-/-*^; ^###^ *P* < .001 *Tph2* ^*+/-*^ vs. *Tph2* ^*-/-*^. Please note that the error bars in B are too small to be visible.

As expected, all developmental milestones, namely body temperature regulation (*F*_2,52_ = 9.220, *P* < .001; Figure 4A) and body weight gain (*F*_2,52_ = 444.921, *P* < .001; Figure 4B), as well as one of the two somatosensory reflexes assessed, namely surface righting (*F*_2,50_ = 25.517, *P* < .001), but not vertical screen holding (*F*_2,50_ = 1.779, *P* = .179), varied with age. Body temperature and body weight increased with age, whereas the time needed for surface righting decreased with age (data not shown in detail).

### Developmental milestones and somatosensory reflexes - genotype

Body temperature regulation differed between genotypes (main effect genotype: *F*_2,26_ = 13.415, *P* < .001; interaction genotype × development: *F*_4,52_ = 3.731, *P* = .010; Figure 4A). Differences in body temperature emerged with development. While genotypes did not differ on PND3 (all *P* values > .050), prominent differences were detected on PND6 and PND9. On both days, *Tph2*^*-/-*^ mouse pups had lower body temperatures than *Tph2*^*+/-*^ (*P* = .007 and *P* = .002) and *Tph2*^*+/+*^ (*P* = .001 and *P* < .001) mouse pups, with the latter not differing from each other (*P* = .384 and *P* = .595). Even more prominent genotype differences were obtained for body weight gain (main effect genotype: *F*_2,26_ = 113.319, *P* < .001; interaction genotype × development: *F*_4,52_ = 153.783, *P* < .001; Figure 4B). From PND3 onward, *Tph2*^*-/-*^ mouse pups had lower body weights than *Tph2*^*+/-*^ (*P* < .001, *P* < .001, and *P* < .001) and *Tph2*^*+/+*^ (*P* < .001, *P* < .001, and *P* < .001) mouse pups, with the latter not differing from each other (all *P* values > .050). Genotypes did not differ in the two somatosensory reflexes assessed, namely surface righting and vertical screen holding (all *P* values > .050).

### Developmental milestones and somatosensory reflexes - sex

Body temperature regulation differed between sexes in a genotype-dependent manner (main effect sex: *F*_1,26_ = .750, *P* = .394; interaction sex × development: *F*_2,52_ = .902, *P* = .405; interaction genotype × sex: *F*_2,26_ = .544, *P* = .587; interaction genotype × sex × development: *F*_4,52_ = 4.380, *P* = .004). In females, no genotype differences were obtained on PND3 (all *P* values > .050). However, while in female *Tph2*^*+/-*^ and *Tph2*^*+/+*^ mouse pups, body temperature increased with development, no such increase was evident in female *Tph2*^*-/-*^ mouse pups. This led to genotype differences on PND6 and PND9. On both days, female *Tph2*^*-/-*^ mouse pups had lower body temperatures than *Tph2*^*+/+*^ controls (*P* = .025 and *P* = .048), but not *Tph2*^*+/-*^ mouse pups (*P* = .290 and *P* = .067), with the latter not differing from each other (*P* = .173 and *P* = .852). Conversely, in males, body temperatures increased in all three genotypes, but already on PND3, *Tph2*^*-/-*^ mouse pups had body temperatures lower than the ones of *Tph2*^*+/-*^ and *Tph2*^*+/+*^ mouse pups (*P* = .001 and *P* = .028), with the latter not differing from each other (*P* = .052). On PND6, genotypes differed again, with *Tph2*^*-/-*^ mouse pups having lower body temperatures than *Tph2*^*+/-*^ and *Tph2*^*+/+*^ mouse pups (*P* = .011 and *P* = .010), with the latter not differing from each other (*P* = .934). On PND9, no genotype differences were detected (all *P* values > .050). Sex had no effect on body weight gain, surface righting, and vertical screen holding (all P values > .050).

### Correlations between isolation-induced pup ultrasonic vocalizations and developmental milestones

No significant correlations between isolation-induced USV and body weight or body temperature were detected, neither on PND3, PND6, nor PND9 (all *P* values > .100), with only one exception (isolation-induced USV emission rate and body temperature on PND3 in Tph2^+/-^ mouse pups: *r* = .715 and *P* = .013).

## Discussion

In this study, we investigated for the first time whether *Tph2*^*-/-*^ mouse pups display deficits in isolation-induced USV, a widely used behavioral measure to assess developmental delays and communication deficits in rodent models for ASD and linked it to the growth retardation phenotype reported before [31-35]. Consistent with previous studies, very prominent genotype differences were obtained for body weight gain. From PND3 onward, *Tph2*^*-/-*^ mouse pups had clearly lower body weights than *Tph2*^*+/-*^ and *Tph2*^*+/+*^ mouse pups. Delayed growth was accompanied by lower body temperatures in *Tph2*^*-/-*^ mouse pups, possibly reflecting a deficit in thermoregulation. While genotypes did not differ on PND3, prominent differences were detected on PND6 and PND9, with body temperatures being relatively low overall due to the 10-min isolation from dam and littermates. On both days, *Tph2*^*-/-*^ mouse pups had lower body temperatures than *Tph2*^*+/-*^ and *Tph2*^*+/+*^ mouse pups, consistent with our previous reports on deficits in body temperature regulation in adult *Tph2*^*-/-*^ mice [31]. Despite the clear effects on body weight gain and body temperature regulation, the two somatosensory reflexes assessed, namely surface righting and vertical screen holding, were not affected by genotype. This is in line with a recent study by Kane et al. [33] not reporting any differences in surface righting and forepaw grasping, but in other somatosensory reflexes not related to the ones determined in the present study, such as air righting and negative geotaxis. The latter is consistent with the fact that 5-HT is important for the modulation of early motor responses [45], and a more detailed assessment of the development of early motor functions in *Tph2*^*-/-*^ mouse pups appears therefore to be a promising future study.

So far, the reasons for the delayed growth in *Tph2*^*-/-*^ mice remain unknown. We reported that impaired thermoregulation and a dysfunction of the hypothalamo-pituitary-adrenal axis are not the primary causes of the growth retardation [35]. Also, dys-/hypophagia could be ruled out as *Tph2*^*-/-*^ mouse pups were reported to display normal suckling activity and had filled milk pouches [35], despite 5-HT being implicated in the regulation of the early suckling response [46]. Interestingly, during prenatal development, we did not see differences in body weight in *Tph2*^*-/-*^ mice and *Tph2*^*+/+*^ controls [35], indicating that the developmental delay emerges after birth. Starting from about PND3, *Tph2*^*-/-*^ mouse pups are smaller, have soft skin, and differ in body weight [31-35]. Here, we now show for the first time that *Tph2*^*-/-*^ mouse pups display a clear deficit in the emission of isolation-induced USV, exactly during growth retardation onset. On both PND3 and PND6, *Tph2*^*-/-*^ mouse pups emitted fewer isolation-induced USV than *Tph2*^*+/+*^ controls. The inverted U-shaped developmental call emission pattern that is typically seen in mouse pups is clearly detectable in *Tph2*^*+/+*^ controls but less prominent in *Tph2*^*-/-*^ mouse pups. The absence of genotype effects on the latency to start calling and call duration indicates that the lack of brain 5-HT primarily affected call repetition rate, i.e., numbers of isolation-induced USV per time interval. While an overall quantitative comparison of the averages for peak frequency, peak amplitude, and frequency modulation did not reveal additional genotype differences, a recently developed more detailed analysis using density plots for individual isolation-induced USV [10] helped to detect 5-HT effects on call clustering on PND6. Confirming the results of the detailed analysis [10] of data obtained in a previous study [42], two clusters were identified in *Tph2*^*+/+*^ controls, with most isolation-induced USV either being characterized by peak frequencies between 60 to 70 and 80 to 110 kHz. In *Tph2*^*-/-*^ mouse pups, two similar clusters were obtained, yet they were not as clearly segregated as in the *Tph2*^*+/+*^ controls. Also, the most common peak frequency was below 60 kHz and therefore clearly lower than in the *Tph2*^*+/+*^ controls. A substantial number of isolation-induced USV emitted by the *Tph2*^*-/-*^ mouse pups were further characterized by comparatively low peak amplitudes*.* Finally, an additional sequential analysis of the durations of subsequent isolation-induced USV indicated that the call emission pattern is not random in all *Tph2*^*+/+*^ controls tested, since the durations of given isolation-induced USV could be predicted by the durations of the previous ones. Evidence for such a non-random call emission pattern was also obtained in *Tph2*^*-/-*^ mouse pups but in fewer cases. A similarly distorted sequential organization was recently reported the first time for a genetic mouse model for ASD, the *Shank1* deficient mouse [10], which displays a variety of behavioral alterations with relevance to ASD [42,47-49] (for a call emission pattern analysis in *Shank2* deficient mice, see [50]). Sex was found to have a minor effect on the emission of isolation-induced USV. Specifically, a sex difference was obtained for call duration but not call number, with females emitting calls with longer durations than males, irrespective of genotype. The comparatively minor effect of sex is consistent with the vast majority of the literature [51-54] (but see [55]). Interestingly, however, we observed longer call durations in females than in males in a number of our previous studies on pup isolation-induced USV, at least in wildtype mice from various strains, including C57BL/6J and 129S6/SvEvTac [42,44,56].

It is unlikely that the observed reduction in isolation-induced USV in *Tph2*^*-/-*^ mouse pups is simply due to the deficits in thermoregulation caused by a lack of brain 5-HT. This is because the developmental trajectories of the deficits in isolation-induced USV and thermoregulation do not match. For instance, a marked reduction in isolation-induced USV was already evident on PND3, at which differences in body temperature were not yet detected. Also, on PND9, when deficits in thermoregulation were most prominent, isolation-induced USV did not differ between genotypes. The same might be true for body weight. Again, most prominent genotype differences in body weight were evident on PND9, when no genotype differences in isolation-induced USV were detected. While deficits in thermoregulation and body weight gain are therefore unlikely to be responsible for the observed reduction in isolation-induced USV in *Tph2*^*-/-*^ mouse pups, the opposite might be true, namely that the reduced USV levels result in thermoregulatory deficits and delayed body weight gain. In rats, it was shown that surgical devocalization, resulting in a complete lack of isolation-induced USV, slowed rewarming of hypothermic pups [57], yet additional experiments are needed to test whether the reduction in USV emission displayed by *Tph2*^*-/-*^ mouse pups is sufficient to cause similar thermoregulatory deficits. In such future experiments, it would be ideal to assess changes in body temperature continuously during isolation from dam and littermates in order to link body temperature changes and the emission of isolation-induced USV over time, for instance, by using a fine rectal thermocouple, as done by Cummings et al. [58]. By means of the present approach, no robust correlations between the two measures were detected.

Isolation-induced USV in mouse pups serve an important communicative function in coordinating mother-pup interactions. Specifically, pup isolation-induced USV elicit maternal behavior, such as search and retrieval behavior, as repeatedly shown in playback experiments [44,59-63]. It was further shown that dams can distinguish between different USV types, and that they prefer certain types over others if given the choice, indicating that acoustic parameters, such as call duration, peak frequency, and peak amplitude, affect the communicative value of isolation-induced USV [44,60,61,63]. Because *Tph2*^*-/-*^ mouse pups emit not just fewer isolation-induced USV, but also USV that are characterized by a lower amplitude level, as compared to *Tph2*^*+/+*^ controls, it appears likely that *Tph2*^*-/-*^ mouse pups are less efficient in attracting mothers and inducing maternal care. Impaired ultrasonic communication in *Tph2*^*-/-*^ mouse pups might therefore explain the growth retardation phenotype emerging around PND3. It is consistent with that view that *Tph2*^*-/-*^ mice slowly catch up in body weight during later developmental periods when the maternal caregiving behavior is becoming less crucial for the further development of the offspring [31,33-35].

Our finding of reduced isolation-induced USV in *Tph2*^*-/-*^ mouse pups lacking brain 5-HT is consistent with the view that the 5-HT system is strongly involved in the regulation of ultrasonic communication in pups. In particular, it is in line with the observation that the reduction of central 5-HT levels by means of various approaches, including the neurotoxin 5,7-DHT, the 5-HT synthesis inhibitor PCPA, and repeated MDMA treatment, blocks isolation-induced USV in rat pups [11,12]. Conversely, however, also increased 5-HT levels following acute MDMA and SSRI treatment, including fluoxetine, paroxetine, and zimeldine, were associated with reduced isolation-induced USV in mice [24] and rats [20,21,25-29]. Likewise, some tricyclic antidepressants inhibiting 5-HT reuptake, such as clomipramine, also inhibit pup isolation-induced USV, while for others, no or even opposite effects were reported [20,21,25-27,29,64-67]. Yet, all of these drugs have several off-target effects besides affecting the 5-HT transporter system in the CNS. Considering that all these pharmacological manipulations resulted only in a temporal and/or partial change in 5-HT content, but still profoundly altered isolation-induced USV emission, it might come as a surprise that a complete lack of brain 5-HT does not result in a complete lack of pup USV. Furthermore, it still needs to be elucidated which 5-HT receptors in particular modulate isolation-induced USV emission, despite comparative pharmacological studies targeting various 5-HT receptors [14-23] and some studies in genetic mouse models pointing towards the importance of 5-HT1A and 5-HT1B receptors in modulating isolation-induced USV [68-72].

Our present findings further fit nicely to known behavioral phenotypes of *Tph2*^*-/-*^ mice. For instance, they display clearly decreased levels of anxiety-related behavior in elevated plus maze, novelty suppressed feeding, and light-dark box [36,37]. Isolation-induced USV were repeatedly linked to anxiety, particularly in pharmacological studies, and it is believed that high call emission rates reflect high anxiety levels. For instance, benzodiazepines, such as diazepam and chlordiazepoxide, but also partial 5-HT1A agonists, including buspirone, consistently led to a dose-dependent decrease in isolation-induced USV [12,15,19-21,23,25-27,64-67,73]. Thus, the greater the amount of isolation-induced USV in pups, the greater the anxiety level observed in the mouse model. Our present findings clearly support this existing view and decreased calling after isolation in *Tph2*^*-/-*^ mouse pups might be correlated with reduced anxiety-related behavior in adulthood [36,37]. However, it has to be noted that the reduced level of anxiety-related behavior in elevated plus maze, novelty suppressed feeding, and light-dark box displayed by *Tph2*^*-/-*^ mice is possibly caused by enhanced impulsivity, as suggested by overall reduced response latencies [36,37]. Also, in the social context, increased impulsivity is evident. *Tph2*^*-/-*^ mice were found to display increased aggression, as reflected by both a decrease in the latency to attack intruders and an increase in the number of attacks, paralleled by increased testosterone levels [31,34,36,37,39].

However, such prominent deficits in the social domain are also consistent with the reduction in isolation-induced USV displayed by *Tph2*^*-/-*^ mouse pups. In fact, the obtained evidence for impairment in early mother-infant communication is in accordance with a recent study suggesting that *Tph2*^*-/-*^ mice display deficits with relevance to all ASD core symptoms [33]. In line with its role as a developmental signal, altered 5-HT signaling has been repeatedly associated with neurodevelopmental disorders, most notably ASD [4]. Importantly, in the CNS, 5-HT innervation seems to be transiently increased during infancy. Specifically, Chugani et al. [74,75] showed by positron emission tomography of a tryptophan analog that children with ASD do not display a peak in brain 5-HT synthesis during early development that is seen in healthy controls. Interestingly, this developmental alteration in 5-HT synthesis was found to be linked to deficits in language acquisition [76]. Also, more recently, decreased serotonin transporter (SERT) binding has been reported in children with ASD [77]. In line with these findings, alterations in genes encoding components of the 5-HT system have been repeatedly reported [4], including *TPH2* [78-80] (but see [81-84]), and hence, a number of genetic mouse models for ASD targeted the 5-HT system, with isolation-induced USV being assessed in some of them. For instance, mice carrying a SERT ALA56 mutation, which was originally found in the human *SERT* gene of ASD families, display an increase in 5-HT levels [85] and recapitulate some features of ASD-like behavior, including social dysfunction and altered isolation-induced USV emission rates [85,86]. Likewise, SERT knockout mice were also found to display altered levels of isolation-induced USV [87]. Importantly, changes in 5-HT1A and 5-HT2A receptor activity were observed in the SERT ALA56 model [85]. As the 5-HT1A and 5-HT2A receptor are both involved in the regulation of isolation-induced USV, it is not clear whether the changes observed in the SERT ALA56 are due to altered 5-HT content. Furthermore, mice lacking *Celf6*, a gene identified by translational profiling of 5-HTergic neurons, were found to have lower 5-HT brain levels and to display fewer isolation-induced USV [88]. Finally, in the 15q11-13 duplication model for ASD, in which increased responses to 5-HT2C receptor signaling was observed, the rate of isolation-induced USV is clearly elevated [89]. Yet, in none of these studies was the specific effects of a decrease and/or lack of brain 5-HT on isolation-induced USV studied.

## Conclusions

In summary, *Tph2*^*-/-*^ mouse pups displayed ultrasonic communication impairment during early development that is likely to result in a deficient mother-infant interaction, presumably contributing to their growth retardation phenotype. Moreover, the ultrasonic communication impairment is consistent with other behavioral phenotypes displayed by *Tph2*^*-/-*^ mice and represents a prominent feature relevant to ASD.

### Availability of supporting data

Recordings of ultrasonic vocalizations will be made available using mouseTube, an online platform for sharing ultrasonic vocalization recordings that is currently established by Thomas Bourgeron, Elodie Ey, and Nicolas Torquet from Institut Pasteur, Paris, France.

## Abbreviations

5,7-DHT: 5,7 dihydroxytryptamine
5-HT: 5-hydroxytryptamine, serotonin
5-HTP: 5-hydroxytryptophan
ASD: autism spectrum disorders
CNS: central nervous system
PCPA: para-chlorophenylalanine
PND: postnatal day
SERT: serotonin transporter
SSRI: selective serotonin reuptake inhibitors
TPH: tryptophan hydroxylase
USV: ultrasonic vocalizations
